# Trainee advocacy for medical education on the care of people with intellectual and/or developmental disabilities: a sequential mixed methods analysis

**DOI:** 10.1186/s12909-024-05449-4

**Published:** 2024-05-03

**Authors:** Lauren Clarke, Nora O’Neill, Binisha Patel, Samantha Steeman, Gabrielle Segal, Sylvia Bereknyei Merrell, Michael A. Gisondi

**Affiliations:** 1 Stanford University School of Medicine, 291 Campus Drive, Stanford, CA 94305 USA; 2 Yale School of Medicine, New Haven, CT USA; 3 University of Texas Medical Branch John Sealy School of Medicine, Galveston, TX USA; 4 Department of Pediatrics, Stanford University School of Medicine, Stanford, CA USA; 5 Department of Emergency Medicine and Principal, The Precision Education and Assessment Research Lab, Stanford University School of Medicine, Stanford, CA USA

**Keywords:** Trainee advocacy, Curricular change, Intellectual and developmental disability

## Abstract

**Background:**

Medical trainees (medical students, residents, and fellows) are playing an active role in the development of new curricular initiatives; however, examinations of their advocacy efforts are rarely reported. The purpose of this study was to understand the experiences of trainees advocating for improved medical education on the care of people with intellectual and/or developmental disabilities.

**Methods:**

In 2022–23, the authors conducted an explanatory, sequential, mixed methods study using a constructivist paradigm to analyze the experiences of trainee advocates. They used descriptive statistics to analyze quantitative data collected through surveys. Participant interviews then yielded qualitative data that they examined using team-based deductive and inductive thematic analysis. The authors applied Kern’s six-step approach to curriculum development as a framework for analyzing and reporting results.

**Results:**

A total of 24 participants completed the surveys, of whom 12 volunteered to be interviewed. Most survey participants were medical students who reported successful advocacy efforts despite administrative challenges. Several themes were identified that mapped to Steps 2, 4, and 5 of the Kern framework: “Utilizing Trainee Feedback” related to Needs Assessment of Targeted Learners (Kern Step 2); “Inclusion” related to Educational Strategies (Kern Step 4); and “Obstacles”, “Catalysts”, and “Sustainability” related to Curriculum Implementation (Kern Step 5).

**Conclusions:**

Trainee advocates are influencing the development and implementation of medical education related to the care of people with intellectual and/or developmental disabilities. Their successes are influenced by engaged mentors, patient partners, and receptive institutions and their experiences provide a novel insight into the process of trainee-driven curriculum advocacy.

**Supplementary Information:**

The online version contains supplementary material available at 10.1186/s12909-024-05449-4.

## Background

The Carnegie Foundation report published in 2010 called for increased individualization of the learning process and decreased rigidity in medical training due to the evolving complexities of science, society, and the medical profession [1]. Trainees (medical students, residents, and fellows) have played an instrumental role in expediting the individualization of their education. A substantial amount of curricular reform in US medical schools has come at the demands of trainees: especially as it relates to topics such as social determinants of health, health equity, diversity, anti-racism, global health, and climate change [2–7]. Trainees have also begun to advocate for improved accessibility and quality of education about the care of people with intellectual and/or developmental disabilities (IDD), a topic rarely addressed in medical eduation[8].

IDDs are a group of conditions characterized by significant limitations in cognitive functioning and adaptive behavior that begin during an individual’s developmental period. These conditions often have a wide range of etiologies and presentations [9]. While physicians of all specialties will provide care to people with IDD, research has shown that most physicians are uncomfortable doing so [10–13]. Lack of physician knowledge, skills, and confidence are major barriers to quality care for people with IDD [14].

Curricular interventions have been shown to be effective at improving physician attitudes toward people with IDD [15]. And while there is currently no requirement that US medical schools teach their students to care for people with IDD [16], some institutions have implemented effective educational initiatives on this topic [17–19]. At many of these institutions, trainees are playing a major role in advocating for the inclusion of this content in their curricula; however, descriptions of their experiences with the process of curriculum advocacy are lacking [8].

In this study, we aimed to understand the experiences and processes of trainee-driven curricular advocacy through the lens of medical students, residents, and fellows advocating for new or improved medical education on the care of individuals with IDD. For the purpose of this manuscript, we use the term *trainee advocates* to describe trainees seeking changes to a curriculum and refer to their efforts and processes as *trainee-driven curriculum advocacy*.

## Methods

### Study design

Using a constructivist paradigm [20], we conducted an explanatory, sequential, mixed methods study of medical trainees advocating for curricular reform related to the care of people with IDD [21]. Our purpose was to gain a better understanding of trainees’ experiences with the processes of curriculum advocacy. To do so, we developed a survey instrument and draft interview guide that queried participants about their curriculum advocacy work. We then collected surveys for an initial period of 8 weeks, after which we performed preliminary data analyses to ensure that the final interview guide included the core concepts reflected in the survey responses. After this analysis, the team determined that the draft interview guide addressed these core concepts and that no changes to the guide were required. At this point, both survey and interview data were collected concurrently until the end of the study period. We found that only a portion of the data collected from our surveys and interviews were relevant to the study objective, and for brevity, we report only those data in this manuscript. We used Kern’s six-step approach to curriculum development as our conceptual framework for analyzing and reporting our results [22].

### Study setting and population

We conducted this study from July 2022 to February 2023 at Stanford University School of Medicine, a large research-based medical school in the US. Eligible participants included US allopathic or osteopathic medical students, residents, or fellows who self-identified as advocates for improving medical education regarding the care of people with IDD.

### Survey and interview guide development

There are currently no published surveys that capture the experiences of trainees advocating for changes to their curriculum. We thus developed a new survey tool and interview guide for this study based on literature review, expert opinion, and the personal experiences of one study author (LC) who is a trainee advocate for IDD-focused medical education. The survey tool and interview guide were developed primarily by LC, who worked with other students and a faculty expert on survey design to perform iterative reviews of the survey tool and interview guide for both content and structure. LC then piloted the survey tool and interview guide among a group of trainee advocates in other fields for item generation, survey functionality, matching of item content to construct, optimal item phrasing, and overall quality control. After trainee feedback was incorporated, four faculty members with expertise in IDD and medical education provided additional feedback on content optimization. After an initial 8-week data collection period, we conducted a preliminary analysis and determined that no changes to the draft interview guide were required. The survey tool (Additional File 1) and interview guide (Additional File 2) are published alongside this manuscript.

### Sampling and data collection

We distributed the survey electronically using Qualtrics (Qualtrics Software Company, Seattle, Washington) during a six-month period from July 2022 – December 2022. We utilized maximum variation sampling strategies to ensure broad participation from a variety of institutions across the US to increase the external validity of the study [23]. To ensure trainee anonymity, their specific training institution(s) were not elicited: institutional variation was instead measured by the geographic location of training. Initial participant recruitment consisted of a solicitation message through email listservs and social media accounts of The American Academy of Developmental Medicine and Dentistry (AADMD) and The Alliance for Disability in Health Care Education (ADHCE). We selected these national professional societies given their focus on improving the health of people with IDD. This initial recruitment resulted in a majority medical student population. To recruit more residents and fellows, we provided program coordinators at all accredited Neurodevelopmental Disabilities residency programs (5 programs with 24 total residents) and Developmental-Behavioral Pediatrics fellowship programs (36 programs with 153 total fellows) with information about the study and asked them to send the information to their trainees [24, 25]. We collected the program coordinators’ contact information using the American Medical Association FREIDA program database. We also used snowball sampling to increase our sample size and target individuals who are likely to be involved in IDD-focused curriculum advocacy [26].

In the survey tool, we asked participants to provide their email addresses if they were willing to be interviewed for the qualitative arm of the study. After an initial 8-week period of data collection and preliminary analysis, LC conducted one-on-one interviews with a subset of the participants virtually using Zoom (Zoom Video Communications, Inc., San Jose, California) over an 11-week period from August 2022 to December 2022. All participants who provided their email addresses were contacted for an interview. Interview recruitment concluded when thematic saturation (a recurrence of participant statements) was reached and no new themes were identified [27]. All interviews were audio-recorded and transcribed using Zoom. LC edited these transcriptions prior to analysis to ensure accurate representation and the removal of identifying information.

### Quantitative data analysis

We analyzed the survey data using Microsoft Excel (Microsoft Corporation, Redman, Washington) and we performed descriptive analyses to summarize the findings for each measure. Trainees responded to the survey items on a five-point Likert-like scale that ranged from “strongly disagree” (1 point) to “strongly agree” (5 points), and we calculated the mean value for each question. If study participants were advocates during both medical school and residency/fellowship, they were prompted to answer each set of questions twice: one time for each stage of their training. This means that an individual participant could have contributed as many as two entries within the broader dataset.

### Qualitative data analysis

There is no previously developed conceptual framework for trainee-driven curriculum advocacy; however, Kern’s six-step approach is a widely accepted framework for curricular development in medical education and can be used to organize the data collected through this study [22]. Therefore, we used a team-based deductive and inductive in vivo analysis of all interview transcripts. We mirrored Braun and Clark’s stepwise approach to thematic analysis [28]. LC, NO, BP, SS, and GS used an iterative process to identify and modify codes [29]. All investigators agreed with the coding schema through an inter-rater agreement and negotiation process prior to the discussion of potential themes [30]. We then implemented a team-based approach (LC, NO, BP, SS, GS, and MG) for the identification and naming of themes [31]. We used Dedoose (SocioCultural Research Associates, Manhattan Beach, California) to facilitate qualitative analysis.

### Reflexivity

It is important to note that members of the research team have personal and/or professional experience with the work being discussed in this study. LC, NO, BP, SS, and GS are all medical students who are passionate about improving IDD training within their own institutions. While we strove to be unbiased in the processes of data collection and analysis and took many steps to limit the impact of these biases, our personal and professional experiences likely shaped the lens through which we viewed the data. We conducted frequent structured discussions between team members to examine how our experiences may influence the analysis process. Our team-based approach to coding and thematic analysis aimed to mitigate the biases of individual team members. We took these steps to minimize the influence of personal bias and ensure the accurate presentation and analysis of data [32].

## Results

### Demographics

A total of 24 participants completed the survey (Table 1) and 12 of these participants completed an optional interview (Table 2). Survey participants attended medical school, residency, or fellowship in a total of 19 different US states. Most participants were female (*N* = 19, 79%) and White (*N* = 18, 76%). 71% of participants were medical students (*N* = 17).

**Table 1:**
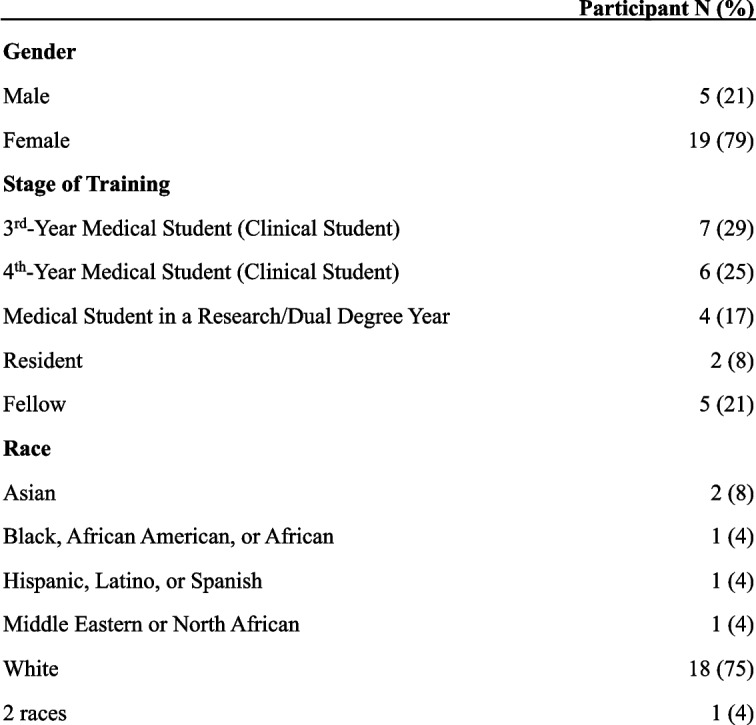
Participant demographics: survey

**Table 2:**
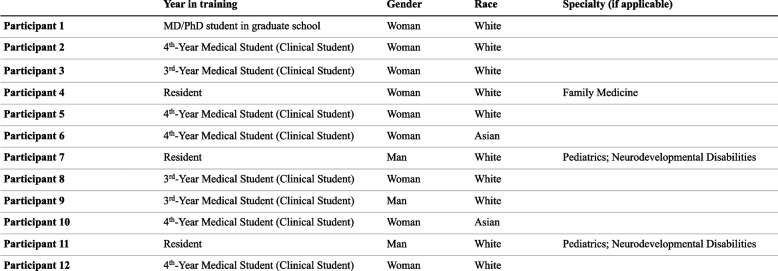
Participant demographics: interviews

### Thematic analysis

We identified five themes in our analysis, which we mapped to Steps 2, 4, and 5 of Kern’s six-step approach to curriculum development [22]. Theme descriptions and representative quotations can be found in Table 3.

**Table 3:**
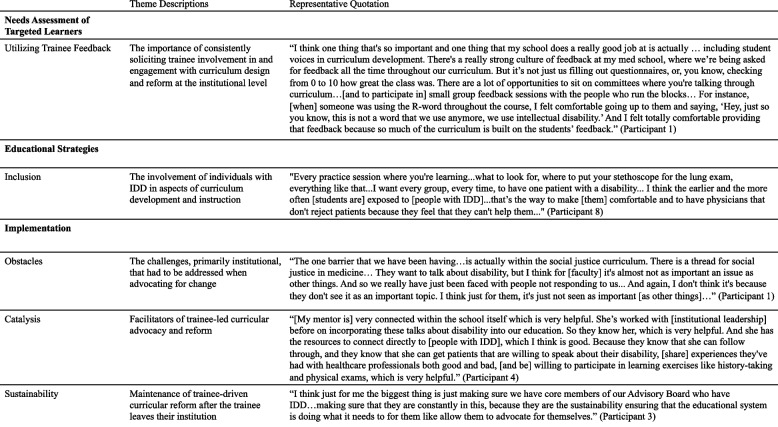
Thematic analysis of participant interviews

### Needs assessment of targeted learners (Kern Step 2)

In the interviews, participants emphasized the importance of soliciting meaningful feedback from trainees regarding their learning and maintaining transparency in regards to how trainee feedback is used to make improvements to the curriculum. We therefore identified the theme of Utilizing Trainee Feedback that mapped well to Kern Step 2 (Table 3).

### Utilizing trainee feedback

Many participants felt that institutions should be more transparent with how trainee feedback is being used to guide curricular development and reform. Participants not only described how institutions could meaningfully engage with trainees about their education but also mentioned ways the feedback process could be improved. For example, Participant 1 (medical student) noted that at their institution “it’s not just us filling out questionnaires, or, you know, checking from 0 to 10 how great the class was. There are a lot of opportunities to sit on committees where you’re talking through curriculum…[and to participate in] small group feedback sessions with the people who run the blocks…” However, it was clear that some participants lacked clarity on how their feedback was being used to improve the curriculum. For example, Participant 3 (medical student) explained that “I always give very specific feedback on how different lectures could include different topics related to [IDD]. But I don’t know if anyone actually reads [it].” Participant 11 (resident) also brought up some of the challenges with the traditional way of soliciting trainee feedback: “I think a lot of times students are encouraged to be on curriculum committees… But a lot of times they either take place during times where we’re not always available…[or if] we are available, it’s after already being pulled in a lot of different directions.”

### Educational strategies (Kern Step 4)

**Fig. 1:**
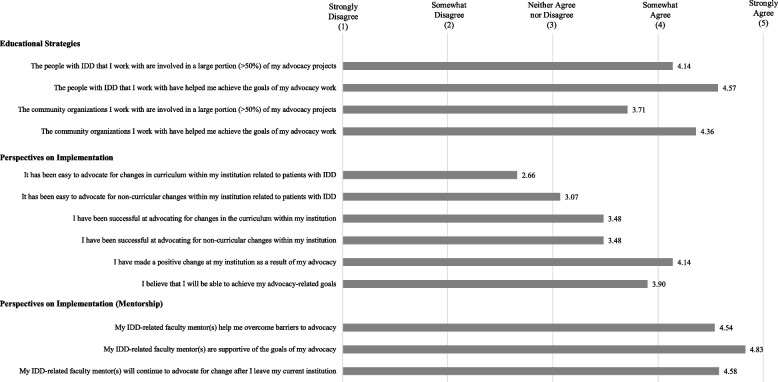
Survey responses

Our results provide novel insight into the educational strategies participants employed in their curricular advocacy work. We found that approximately half of the participants (*N* = 14, 48%) partnered with people with IDD and/or community organizations that work with people with IDD. Participants reported substantial and meaningful engagement of these partners when advocating for curricular change: most participants somewhat or strongly agreed that people with IDD (*N* = 13, 93%) and community organizations (*N* = 13, 93%) helped them achieve their advocacy goals (Fig. 1). We therefore identified the theme of Inclusion that mapped well to Kern Step 4 (Table 3).

### Inclusion

Participants supported the inclusion of people with IDD in both curriculum design and implementation. For example, many participants discussed the importance of gaining direct, firsthand experience working with people with IDD as a part of their training. As Participant 4 (resident) stated, “the biggest fear is the fear of the unknown…[and] the only way you’re going to overcome that barrier is to experience [caring for people with IDD].” That said, some participants described barriers to including people with IDD in their curricular initiatives. For example, when Participant 3 (medical student) was trying to hire people with IDD as standardized patients, they found that the notary process that the school required wasn’t accessible: “You have to go on to the second floor, and the elevator isn't large enough for certain size wheelchairs. We had a student become a notary so that they could get their hiring paperwork done.” Participants also stressed the importance of routinely including people with IDD in the process of curriculum development itself. Participant 6 (medical student) said, “I want [people with IDD] fully integrated into the school…being part of our faculty somehow in terms of curriculum development…I really want them to represent and reflect what we're learning.”

### Implementation (Kern Step 5)

Participants experienced many obstacles to the implementation of IDD-focused curricula at their local institutions. Very few participants somewhat or strongly agreed that it was easy to advocate for curricular changes (*N* = 8, 27.6%) or non-curricular changes (*N* = 13, 44.8%). Despite this, a higher percentage of the participants somewhat or strongly agreed that their advocacy work successfully resulted in direct changes to the curriculum (*N* = 18, 62%) or other non-curricular aspects of their education (*N* = 17, 58.6%). An even higher percentage of participants (*N* = 22, 75%) somewhat or strongly agreed with the statement, “I have made a positive change at my institution as a result of my advocacy work.” And while trainees on average reported that advocating for non-curricular changes was slightly easier than advocating for curricular changes, their perceived success in advocating for curricular and non-curricular changes was identical. Participants also relied heavily on mentors to assist them with curriculum implementation. 83% (*N* = 29) of participants surveyed said that they had identified a primary mentor, and most of the participants said that they “strongly agreed” or “agreed” that their mentors helped them overcome barriers to advocacy (*N* = 15, 52%) and that their mentors were supportive of their advocacy goals (*N* = 20, 69%). Over half of the participants somewhat or strongly agreed that their mentors would continue to advocate for change once the participants were no longer trainees (*N* = 17, 58%) (Fig. 1). We therefore associated three themes with Kern Step 5: Obstacles, Catalysts, and Sustainability (Table 3).

### Obstacles

Participants described obstacles to their work that stemmed from institutional oversight and a lack of faculty expertise. For example, Participant 6 (medical student) recounted, “as [the curriculum] became more present in the community and people recognized the school for [our] program, I think the administration…were honestly scared of just...how big it [had] gotten, the liability of holding something like this at our school.” Participant 7 (resident) also noted that “there is poor understanding by the providers who are providing [IDD-related] education. So many of the Ph.D. professors and lecturers often have little to no interaction with this population…and then on top of that, some of the guest lectures, who maybe are also attendings at the hospital, also are poor in terms of their experience or knowledge on how to care for [people with IDD].” Some students also expressed difficulty with identifying faculty partners. As Participant 9 (medical student) put it, “it’s like trying to find a faculty mentor about homosexuality in the 1970s: it ain't happening.” Participants also experienced unique obstacles associated with advocating for content that isn’t traditionally included in medical education. As Participant 4 (resident) explained, “I was constantly told there’s … no time to fit [new curriculum] in. Which is frustrating because we find time for a lot of other things…so it's frustrating that it's not more of a priority.” Participants also described obstacles that are inherent to the process of trainee-driven curriculum advocacy. For example, Participant 1 (medical student) noted that a major barrier was “…just not always being able to develop curriculum while you're in med school because [medical school is] very stressful.” Participant 1 also pointed out that “a lot of med students…don't have a lot of personal experience doing curriculum development.”

### Catalysts

In contrast to the aforementioned obstacles, participants also identified several catalysts that facilitated their advocacy work. These catalysts for change included faculty champions, mentors, and other trainee advocates. Participant 10 (medical student) attributed their success to “the fact that [faculty] were supportive of all the initiatives.” Participant 4 (resident) also commented on the importance of faculty support when they described their appreciation for faculty “giving me the freedom to explore some [IDD-related] topics with my colleagues. I find that often I get… self-conscious or worried [that IDD-focused curriculum is not the] most applicable to everyone. But [my faculty] have never made me feel that way…And so I'm appreciative that they are open to my topics and want to teach those things.” Mentors also served as catalysts to implementation. For example, Participant 11 (resident) said that their mentor helped them by “know[ing] some of the institutional or policy-specific things that tend to get in the way and [giving] me some pointers on how to work or maneuver around those kinds of barriers.” Participant 8 (medical student) also noted that her mentor is “very connected within the school itself which is very helpful.” Other trainees can also serve as catalysis. For example, Participant 2 (medical student) noted that working with another student allowed them to “be able to keep on being productive, keep things moving, in a project when either one of [us is] busy.” Participant 1 (medical student) had a similar strategy of partnering with classmates: “I think one of the biggest strategies that we had was actually working with medical students who were in different areas or in different years… [This allowed us] to split up that work and be more collaborative, so that we would all at least have a little bit of time to contribute.”

### Sustainability

Many participants expressed concerns over the sustainability of their work. Methods that trainees took to increase the sustainability of their curricular iniatives varied widely, from identifying faculty members and peers who would carry on the advocacy work to ensuring the requirement of IDD-focused curriculum and/or the continued involvement and representation of people with IDD within the curriclum. For example, Participant 3 (medical student) expressed doubt about the sustainability of their work and noted that “having a faculty mentor that is going to be there for an extended period of time would be great. Knowing there [would be] somebody…still advocating for the changes would be huge.” On the other hand, Participant 10 (medical student) noted that she wasn’t worried about the sustainability of her IDD-focused curricular session because it “[has already ran] for 2 years, it’s going to run again this year, and it's designed in a way that I don't need to be there for it to continue running. The course coordinators have all the information and all the documents to do so.”

## Discussion

The results of our study provide novel insight into the process of trainee-driven curricular advocacy and the experiences of trainee advocates. Trainee demand for curriculum reform is nothing new: there is ample evidence and myriad anecdotes of trainees requesting new curricula and institutions responding positively or not [3]. However, the efforts and deliberate actions taken by trainees to revise their own curricula are rarely described. Our findings provide insights into the process of trainee-driven curriculum advocacy: a topic unexplored in the literature to-date. We learned that successful trainee advocates achieve their goals by forming strategic partnerships, teaming with one another and members of the community, navigating institutional barriers through faculty guidance, leveraging the political capital of faculty mentors, building succession plans, and deeply engaging in curriculum feedback. Our participants told rich stories of their experiences, detailing the time, strategy, politics, negotiations, failures, and grit inherent to trainee-driven curriculum advocacy. Though the focus of our study was advocacy for IDD education, our work can serve more broadly as a roadmap for trainee advocates and their mentors/institutions to better understand the process of trainee-driven curriculum advocacy with potential application in other fields not well represented within medical education [2, 4, 8].

In discussing different educational strategies participants employed when designing IDD-focused curricula, they emphasized the importance of gaining experience working directly with people with IDD: an educational strategy supported by the literature [33]. Through these direct experiences, medical trainees can gain confidence in caring for people with IDD while developing targeted communication and physical exam skills [18, 34]. Many participants also stressed the importance of including individuals with IDD in the development of curricular materials. Other fields have similarly started to integrate patients’ perspectives into the development of educational material and the teaching of medical students, which has been positively received by both patients and students [35]. As the majority of trainee-driven advocacy focuses on topics related to social determinants of health and/or the needs of marginalized populations [2–5, 8], trainee advocates must be provided with opportunities to meaningfully engage with community members. It is also critical that educational spaces and experiences are accessible to community members, as this will help ensure meaningful participation and engagement of all relevant stakeholders.

In discussing the process of trainee-driven curriculum implementation, participants identified many obstacles. While some of these obstacles were inherent to the process of trainee-driven advocacy, such as having to balance the demands of training with those of advocacy work, others alluded to potential institutional shortcomings. And while participants identified and took advantage of various catalysts that facilitated their advocacy efforts, the results of our research demonstrate the need for formalized institutional support of trainee-driven curriculum advocacy. Trainees may also perceive initiating curricular change as more difficult than non-curricular change, further highlighting the importance of support specific to curricular advocacy. This support would be especially helpful in ensuring the sustainability of trainee-driven advocacy, which has been previously cited as a unique barrier to this work [36]. Though there are some reports of such programs at institutions such as John Hopkins University [37] and The University of Illinois College of Medicine at Chicago [38], our data suggest that these programs do not exist at every institution. But our data do suggest that trainees not only want to be engaged with their curriculum and be given meaningful opportunities to provide feedback on their learning but also want increased clarity regarding how that feedback is being used to improve the curriculum. Data also shows that faculty members who participated in a formalized mentorship program viewed this work as meaningful [39]. Faculty resistance has been cited as a barrier to curricular reform even if it doesn’t originate at the student level [40], suggesting that addressing potential faculty-level resistance to change could have positive impacts beyond trainee advocacy. With trainee-driven advocacy playing an increasingly key role in curriculum reform, institutions should ensure they are supporting these advocates and engaging all learners in the ongoing process of curricular improvement.

National organizations can also play a role in supporting trainee advocates in overcoming some of the aforementioned barriers. While the present study demonstrated that local mentors played a key role in helping students navigate this process, not all participants were able to identify faculty mentors for IDD curriculum reform. And given the fact that trainee-driven advocacy spans a wide variety of fields, topics, and special populations, trainees may not always be able to identify mentors within their home institution. One solution for this lack of mentorship is for national advocacy organizations to offer affordable and accessible opportunities for trainees to meet and engage with faculty leaders on a national level. Another is for these organizations to assist with facilitating the identification of faculty at a trainee’s home institution who can mentor students and assist in the process of curricular advocacy. The mentor/mentee relationships built through these organizations have the potential to not only support trainees in their current curricular advocacy efforts but also turn into life-long relationships that promote the development of the future generation of leaders within the field.

### Limitations

A few limitations must be considered when interpreting the results of this study. As with all survey-based research, our study has the potential to be impacted by recall bias or social desirability bias. To limit the impact of these biases, we collected limited demographic information from participants to ensure that they felt secure in their ability to talk freely about their experiences without the risk of identification. We also aimed to reduce these biases by designing neutral interview questions based on the survey results, performing highly structured interviews, probing based on trainees’ initial responses, and conducting one-on-one interviews rather than focus groups. It is also important to note that our study population consisted only of physician trainees (medical students, residents, and fellows), which means that the findings are not generalizable to trainee advocacy within other fields of healthcare. Regarding participant demographics, we recognize that most self-identified as white women. Therefore, our data does not accurately represent the challenges that trainees of other racial identities may face while navigating the process of curriculum advocacy. Future research should ensure the perspectives of these trainees are captured, and trainees of all racial/ethnic backgrounds should be supported as curriculum advocates. This will help ensure that curricula developed through trainee-driven processes are intersectional in nature and accurately represent the needs of diverse sets of students and patients. It should also be noted that the researchers involved in this project identify as trainee curricular advocates, and it is possible that the personal experiences of the research introduced bias into the coding and thematic analysis processes. However, many steps were taken to decrease this risk, including building on quantitative survey results with qualitative interview data, taking a deductive and inductive approach to data analysis, employing a team-based approach to coding and thematic analysis, and utilizing objective quantitative data to inform the qualitative analysis.

## Conclusions

In this mixed methods study, medical trainees involved in IDD-focused curriculum reform offered their perspectives on the process of trainee-driven curriculum advocacy. The current lack of sufficient medical training related to caring for people with IDD, particularly that which is informed and created by people with IDD themselves, is a glaring reminder of the work that needs to be done in this space. The perspectives shared by trainee advocates, often in close partnership with individuals with IDD, provide valuable insights into the process of trainee-driven curriculum advocacy. We hope that this not only lays the foundation for future research on the process of trainee-driven advocacy but also encourages institutions to support their trainees who are involved in these efforts.

### Supplementary Information

**Additional file 1:** Microsoft Word document. Copy of survey tool.

**Additional file 2:** Microsoft Word document. Copy of interview guide.

## Data Availability

The datasets generated and/or analyzed during the current study are not publicly available due to the potentially sensitive nature of the qualitative data collected and to ensure complete anonymity of student participants but are available from the corresponding author on reasonable request.

## Abbreviation

IDD: Intellectual and/or Developmental Disabilities

## References

[1] Cooke M, Irby D, O'Brien B (2010). Educating physicians: a call for reform of medical school and residency.

[2] Afolabi T, Borowsky HM, Cordero DM, Paul DW, Said JT, Sandoval RS (2021). Student-led efforts to advance anti-racist medical education. Acad Med.

[3] Forrest LL, Geraghty JR (2022). Student-led initiatives and advocacy in academic medicine: empowering the leaders of tomorrow. Acad Med.

[4] Goshua A, Gomez J, Erny B, Burke M, Luby S, Sokolow S (2021). Addressing climate change and its effects on human health: a call to action for medical schools. Acad Med.

[5] Spivey Provencio SJ, Singh Y, Roy A (2022). medical student-led effort to prioritize health equity and diversity in preclinical case-based learning. Acad Med.

[6] Hashmi SS, Saad A, Leps C, Gillies-Podgorecki J, Feeney B, Hardy C (2020). A student-led curriculum framework for homeless and vulnerably housed populations. BMC Med Educ.

[7] Nguyen AX-L, Xiang L, Chhibber R, Blanchard H, Tikhonova S, Zafran H (2023). Student-led interprofessional global health course: learning impacts during a global crisis. BMC Med Educ.

[8] Clarke L (2023). The need to include intellectual/developmental disability in medical school curriculum: the perspective of a student advocate. J Intellect Dev Disabil.

[9] Lee K, Cascella M, Marwaha R (2022). Intellectual Disability. [Updated 2022 Sep 21].

[10] Iezzoni LI, Rao SR, Ressalam J, Bolcic-Jankovic D, Agaronnik ND, Donelan K (2021). physicians’ perceptions of people with disability and their health care. Health Aff.

[11] Campbell EG, Rao SR, Ressalam J, Bolcic-Jankovic D, Lawrence R, Moore JM (2022). Caring for adults with significant levels of intellectual disability in outpatient settings: results of a national survey of physicians. Am J Intellect Dev Disabil.

[12] Wilkinson J, Dreyfus D, Cerreto M, Bokhour B (2012). "Sometimes I feel overwhelmed": educational needs of family physicians caring for people with intellectual disability. Intellect Dev Disabil.

[13] Campbell EG, Rao SR, Ressalam J, Bolcic-Jankovic D, Lawrence R, Moore JM (2023). Caring for Adults With Significant Levels of Intellectual Disability in Outpatient Settings: Results of a National Survey of Physicians. Am J Intellect Dev Disabil.

[14] Doherty AJ, Atherton H, Boland P, Hastings R, Hives L, Hood K (2020). Barriers and facilitators to primary health care for people with intellectual disabilities and/or autism: an integrative review. BJGP Open.

[15] Ryan TA, Scior K (2014). Medical students' attitudes towards people with intellectual disabilities: a literature review. Res Dev Disabil.

[16] Functions and Structure of a Medical School: Standards for Accreditation of Medical Education Programs Leading to the MD Degree: Liaison Committee on Medical Education (LCME). Available from: www.lcme.org/publications/.

[17] Coret A, Boyd K, Hobbs K, Zazulak J, McConnell M (2018). Patient narratives as a teaching tool: a pilot study of first-year medical students and patient educators affected by intellectual/developmental disabilities. Teach Learn Med.

[18] Long-Bellil LM, Robey KL, Graham CL, Minihan PM, Smeltzer SC, Kahn P (2011). Teaching medical students about disability: the use of standardized patients. Acad Med.

[19] Clarke L, Tabor HK (2023). The impact of inclusion: Improving medical student confidence in caring for adults with intellectual disabilities through an interactive, narrative-based session. J Intellect Dev Disabil.

[20] Teherani A, Martimianakis T, Stenfors-Hayes T, Wadhwa A, Varpio L (2015). Choosing a qualitative research approach. J Grad Med Educ.

[21] Creswell JW, Plano Clark VL. Designing and Conducting Mixed Methods Research. 3rd ed. Thousand Oaks, CA: SAGE; 2018.

[22] Thomas PA, Kern DE, Hughes MT (2016). Curriculum Development for Medical Education : A Six-Step Approach.

[23] Patton M. Qualitative Research and Evaluation Methods. 4th ed. Thousand Oaks: Sage; 2014.

[24] The National Resident Matching Program (NRMP). Advance Data Tables: 2023 Main Residency Match. 2023.

[25] The National Resident Matching Program (NRMP). Results and Data: Specialties Matching Service. 2023.

[26] Palinkas LA, Horwitz SM, Green CA, Wisdom JP, Duan N, Hoagwood K (2015). Purposeful sampling for qualitative data collection and analysis in mixed method implementation research. Adm Policy Ment Health.

[27] Saunders B, Sim J, Kingstone T, Baker S, Waterfield J, Bartlam B (2018). Saturation in qualitative research: exploring its conceptualization and operationalization. Qual Quant.

[28] Braun V, Clarke V (2006). Using thematic analysis in psychology. Qual Res Psychol.

[29] Giesen L, Roeser A (2020). Structuring a team-based approach to coding qualitative data. Int J Qual Methods.

[30] Campbell JL, Quincy C, Osserman J, Pedersen OK (2013). Coding in-depth semistructured interviews: problems of unitization and intercoder reliability and agreement. Sociol Methods Res.

[31] MacQueen KM, McLellan E, Kay K, Milstein B (1998). Codebook development for team-based qualitative analysis. Cam Journal.

[32] O’Brien BC, Harris IB, Beckman TJ, Reed DA, Cook DA (2014). Standards for Reporting Qualitative Research: A Synthesis of Recommendations. Acad Med.

[33] Tracy J, Iacono T (2008). People with developmental disabilities teaching medical students – does it make a difference?. J Intellect Dev Disabil.

[34] Thomas B, Courtenay K, Hassiotis A, Strydom A, Rantell K (2014). Standardised patients with intellectual disabilities in training tomorrow's doctors. Psychiatr Bull (2014).

[35] Dijk SW, Duijzer EJ, Wienold M (2020). Role of active patient involvement in undergraduate medical education: a systematic review. BMJ Open.

[36] Fletcher A, Chen BY, Benrimoh D, Shemie S, Lubarsky S (2018). Lessons learned from a student-driven initiative to design and implement an organ and tissue donation course across Canadian medical schools. Perspect Med Educ.

[37] Hsih KW, Iscoe MS, Lupton JR, Mains TE, Nayar SK, Orlando MS (2015). The student curriculum review team: how we catalyze curricular changes through a student-centered approach. Med Teach.

[38] Geraghty JR, Young AN, Berkel TDM, Wallbruch E, Mann J, Park YS (2020). Empowering medical students as agents of curricular change: a value-added approach to student engagement in medical education. Perspect Med Educ.

[39] Roussel D, Gordon PR, Wagner JM, Bardack M, Sardesai MG, Colbert-Getz JM (2020). The learning community faculty experience: how longitudinal relationships with learners enhance work meaning. Perspect Med Educ.

[40] Pock AR, Durning SJ, Gilliland WR, Pangaro LN (2019). Post-Carnegie II curricular reform: a north American survey of emerging trends & challenges. BMC Med Educ.

