# Supplementary material for: Trainee advocacy for medical education on the care of people with intellectual and/or developmental disabilities: a sequential mixed methods analysis
Source: BMC Med Educ. 2024 May 3;24:491. doi: 10.1186/s12909-024-05449-4 (PMC11067383; doi:10.1186/s12909-024-05449-4)
Supplement: Supplementary file 1 — Additional file 1: Microsoft Word document. Copy of survey tool. [file 12909_2024_5449_MOESM1_ESM.docx]

The experience of students and residents advocating for improved medical education in IDD

Demographics

1. What is your current year of training?

- M1
- M2
- M3
- M4
- Other year of medical school (please specify)
- MD/PhD student in graduate school
- Resident
- Fellow

2. What sex were you assigned at birth (ie what is on your original birth certificate)?

- Female
- Male
- Self-Describe
- Prefer not to say

3. What is your gender identity?

- Man
- Woman
- Non-binary
- Self-Describe
- Prefer not to say

4. Which best describes your race? Check all that apply.

- American Indian or Alaska Native (For example: Aztec, Blackfeet Tribe, Mayan, Navajo Nation, Native Village of Barrow (Utqiagvik) Inupiat Traditional Government, Nome Eskimo Community, etc.)
- Asian (For example: Asian Indian, Chinese, Filipino, Japanese, Korean, Vietnamese, etc.)
- Black, African American, or African (For example: African American, Ethiopian, Haitian, Jamaican, Nigerian, Somali, etc.)
- Caribbean (For example: Cuban, Dominican, Jamaican, Guyanese, Trinidadian and Tobagonian, Haitian, etc.)
- Hispanic, Latino, or Spanish (For example: Colombian, Cuban, Dominican, Mexican or Mexican American, Puerto Rican, Salvadoran, etc.)
- Middle Eastern or North African (For example: Algerian, Egyptian, Iranian, Lebanese, Moroccan, Syrian, etc.)
- Native Hawaiian or other Pacific Islander (For example: Chamorro, Fijian, Marshallese, Native Hawaiian, Tongan, etc.)
- White (For example: English, European, French, German, Irish, Italian, Polish, etc.)
- Other race not listed (please specify)
- Prefer not to say

5. What is your age (in years)?

6. What specialty is your residency program in? (ie pediatrics, med-peds, internal medicine, etc.)

7. What specialty was your residency program in? (ie pediatrics, med-peds, internal medicine, etc.)

8. What fellowship are you currently pursuing?

9. Did you perform curriculum advocacy related to intellectual/developmental disabilities (IDD) as a medical student, as a resident/fellow, or as both?

- Only as a medical student (1)
- Only as a resident or fellow (2)
- Both as a medical student and as a resident/fellow (3)

10. In which state or country (if outside of the United States) did you perform your IDD-related advocacy work as a medical student?

▼ Alabama (1) ... Outside of the United States or Canada (53)

11. In which state or country (if outside of the United States) did you perform your IDD-related advocacy work as a resident/fellow?

▼ Alabama (1) ... Outside of the United States or Canada (53)

12. Are you interested in being contacted for a 1hr interview on your IDD-related advocacy experience as a part of this study? If yes, please provide your email. This email will not be linked to survey data and will only be used for the purposes of follow-up communication.

- Yes-please provide email
- No

Educational Strategies

1. Did you enlist the help of external community organizations (Special Olympics, Best Buddies, etc.) in your intellectual/developmental disability (IDD)-related advocacy as a medical student?

- Yes
- No
- Unsure

2. Please rate the following statements as they refer to your experience performing IDD-related curriculum advocacy as a medical student.

|  | Strongly disagree | Somewhat disagree | Neither agree nor disagree | Somewhat agree | Strongly agree |
| --- | --- | --- | --- | --- | --- |
| The community organizations I work with are involved in a large portion (>50%) of my advocacy projects |  |  |  |  |  |
| The community organizations I work with have helped me achieve the goals of my advocacy work |  |  |  |  |  |

3. Did you enlist the help of external community organizations (Special Olympics, Best Buddies, etc.) in your intellectual/developmental disability (IDD)-related advocacy as a resident/fellow?

- Yes
- No
- Unsure

4. Please rate the following statements as they refer to your experience performing IDD-related curriculum advocacy as a resident/fellow.

|  | Strongly disagree | Somewhat disagree | Neither agree nor disagree | Somewhat agree | Strongly agree |
| --- | --- | --- | --- | --- | --- |
| The community organizations I work with are involved in a large portion (>50%) of my advocacy projects |  |  |  |  |  |
| The community organizations I work with have helped me achieve the goals of my advocacy work |  |  |  |  |  |

5. Did you enlist the help of self-advocates (people with intellectual disabilities) in your intellectual/developmental disability (IDD)-related advocacy as a medical student?

- Yes
- No
- Unsure

6. Please rate the following statements as they refer to your experience performing IDD-related curriculum advocacy as a medical student.

|  | Strongly disagree | Somewhat disagree | Neither agree nor disagree | Somewhat agree | Strongly agree |
| --- | --- | --- | --- | --- | --- |
| Self-advocates are involved in a large portion (>50%) of my advocacy projects |  |  |  |  |  |
| The self-advocates I work with have helped me achieve the goals of my advocacy work |  |  |  |  |  |

7. Did you enlist the help of self-advocates (people with intellectual disabilities) in your intellectual/developmental disability (IDD)-related advocacy as a resident/fellow?

- Yes
- No
- Unsure

8. Please rate the following statements as they refer to your experience performing IDD-related curriculum advocacy as a resident/fellow.

|  | Strongly disagree | Somewhat disagree | Neither agree nor disagree | Somewhat agree | Strongly agree |
| --- | --- | --- | --- | --- | --- |
| Self-advocates are involved in a large portion (>50%) of my advocacy projects |  |  |  |  |  |
| The self-advocates I work with have helped me achieve the goals of my advocacy work |  |  |  |  |  |

Implementation (Perspectives)

1. Please rate the following statements as they refer to your work in intellectual/developmental disability (IDD)-related advocacy during medical school.

|  | Strongly disagree | Somewhat disagree | Neither agree nor disagree | Somewhat agree | Strongly agree | N/A |
| --- | --- | --- | --- | --- | --- | --- |
| I have been successful at advocating for changes in the curriculum within my institution |  |  |  |  |  |  |
| I have been successful at advocating for non-curricular changes within my institution |  |  |  |  |  |  |
| It has been easy to advocate for changes in curriculum within my institution related to patients with IDD |  |  |  |  |  |  |
| It has been easy to advocate for non-curricular changes within my institution related to patients with IDD |  |  |  |  |  |  |
| I have made a positive change at my institution as a result of my advocacy |  |  |  |  |  |  |
| I believe that I will be able to achieve my advocacy-related goals |  |  |  |  |  |  |

2. Please rate the following statements as they refer to your work in intellectual/developmental disability (IDD)-related advocacy during your residency/fellowship.

|  | Strongly disagree | Somewhat disagree | Neither agree nor disagree | Somewhat agree | Strongly agree | N/A |
| --- | --- | --- | --- | --- | --- | --- |
| I have been successful at advocating for changes in the curriculum within my institution |  |  |  |  |  |  |
| I have been successful at advocating for non-curricular changes within my institution |  |  |  |  |  |  |
| It has been easy to advocate for changes in curriculum within my institution related to patients with IDD |  |  |  |  |  |  |
| It has been easy to advocate for non-curricular changes within my institution related to patients with IDD |  |  |  |  |  |  |
| I have made a positive change at my institution as a result of my advocacy |  |  |  |  |  |  |
| I believe that I will be able to achieve my advocacy-related goals |  |  |  |  |  |  |

Implementation (Impact of Faculty)

1. Is/Was the leadership at your institution’s school of medicine aware of your advocacy work as a medical student? This refers to faculty/professors outside of your IDD-related mentors.

- Yes
- Unsure
- No

2. Please rate the following statements as they refer to your work in intellectual/developmental disability (IDD)-related advocacy as a medical student.

|  | Strongly disagree | Somewhat disagree | Neither agree nor disagree | Somewhat agree | Strongly agree |
| --- | --- | --- | --- | --- | --- |
| Faculty at my medical school who are not my IDD-related mentor(s) help me overcome barriers to advocacy |  |  |  |  |  |
| Faculty at my medical school who are not my IDD-related mentor(s) are supportive of the goals of my advocacy |  |  |  |  |  |
| Faculty at my medical school who are not my IDD-related mentor(s) will continue to advocate for change after I leave my current institution |  |  |  |  |  |

3. Is/Was the leadership at your institution’s residency program and/or Graduate Medical Education (GME) office aware of your advocacy work as a resident/fellow? This refers to faculty/professors outside of your IDD-related mentors.

- Yes
- Unsure
- No

4. Please rate the following statements as they refer to your work in intellectual/developmental disability (IDD)-related advocacy as a resident/fellow.

|  | Strongly disagree | Somewhat disagree | Neither agree nor disagree | Somewhat agree | Strongly agree |
| --- | --- | --- | --- | --- | --- |
| Faculty at my residency/fellowship program who are not my IDD-related mentor(s) help me overcome barriers to advocacy |  |  |  |  |  |
| Faculty at my residency/fellowship program who are not my IDD-related mentor(s) are supportive of the goals of my advocacy |  |  |  |  |  |
| Faculty at my residency/fellowship program who are not my IDD-related mentor(s) will continue to advocate for change after I leave my current institution |  |  |  |  |  |

Mentorship

1. Have you identified at least one faculty mentor at your home institution who supports your IDD-related advocacy work while a medical student?

- Yes
- Maybe
- No

2. Please rate the following statements as they refer to your primary IDD-related faculty mentor while in medical school.

|  | Strongly disagree | Somewhat disagree | Neither agree nor disagree | Somewhat agree | Strongly agree |
| --- | --- | --- | --- | --- | --- |
| My IDD-related faculty mentor(s) help me overcome barriers to advocacy |  |  |  |  |  |
| My IDD-related faculty mentor(s) are supportive of the goals of my advocacy |  |  |  |  |  |
| I believe that my IDD-related faculty mentor(s) will continue to advocate for change after I leave my current institution |  |  |  |  |  |

3. Have you identified at least one faculty mentor at your home institution who supports your IDD-related advocacy work while a resident/fellow?

- Yes
- Maybe
- No

4. Please rate the following statements as they refer to your primary IDD-related faculty mentor while in residency/fellowship.

|  | Strongly disagree | Somewhat disagree | Neither agree nor disagree | Somewhat agree | Strongly agree |
| --- | --- | --- | --- | --- | --- |
| My IDD-related faculty mentor(s) help me overcome barriers to advocacy |  |  |  |  |  |
| My IDD-related faculty mentor(s) are supportive of the goals of my advocacy |  |  |  |  |  |
| My IDD-related faculty mentor(s) will continue to advocate for change after I leave my current institution |  |  |  |  |  |
