# Supplementary material for: Trainee advocacy for medical education on the care of people with intellectual and/or developmental disabilities: a sequential mixed methods analysis
Source: BMC Med Educ. 2024 May 3;24:491. doi: 10.1186/s12909-024-05449-4 (PMC11067383; doi:10.1186/s12909-024-05449-4)
Supplement: Supplementary file 2 — Additional file 2: Microsoft Word document. Copy of interview guide. [file 12909_2024_5449_MOESM2_ESM.docx]

The experience of students and residents advocating for improved medical education in IDD

Demographics

1. What is your current year of training?

- M1
- M2
- M3
- M4
- MD/PhD student in graduate school
- Resident
- Fellow
- Other (please specify)_______________________________________________

2. What sex were you assigned at birth (ie what is on your original birth certificate)?

- Female
- Male
- Self-Describe
- Prefer not to say

3. What is your gender identity?

- Man
- Woman
- Non-binary
- Self-Describe
- Prefer not to say

4. Which best describes your race? Check all that apply.

- American Indian or Alaska Native (For example: Aztec, Blackfeet Tribe, Mayan, Navajo Nation, Native Village of Barrow (Utqiagvik) Inupiat Traditional Government, Nome Eskimo Community, etc.)
- Asian (For example: Asian Indian, Chinese, Filipino, Japanese, Korean, Vietnamese, etc.)
- Black, African American, or African (For example: African American, Ethiopian, Haitian, Jamaican, Nigerian, Somali, etc.)
- Caribbean (For example: Cuban, Dominican, Jamaican, Guyanese, Trinidadian and Tobagonian, Haitian, etc.)
- Hispanic, Latino, or Spanish (For example: Colombian, Cuban, Dominican, Mexican or Mexican American, Puerto Rican, Salvadoran, etc.)
- Middle Eastern or North African (For example: Algerian, Egyptian, Iranian, Lebanese, Moroccan, Syrian, etc.)
- Native Hawaiian or other Pacific Islander (For example: Chamorro, Fijian, Marshallese, Native Hawaiian, Tongan, etc.)
- White (For example: English, European, French, German, Irish, Italian, Polish, etc.)
- Other race not listed (please specify)
- Prefer not to say

5. What is your age (in years)?

6. What specialty is your residency in? (ie pediatrics, med-peds, internal medicine, etc.)

7. What specialty did you do your residency in? (ie pediatrics, med-peds, internal medicine, etc.)

8. What fellowship are you currently pursuing?

9. Did you perform curriculum advocacy related to intellectual/developmental disabilities (IDD) as a medical student, as a resident/fellow, or as both?

- Only as a medical student
- Only as a resident
- Only as a fellow
- Both as a medical student and as a resident/fellow

10. In which state or country (if outside of the United States) did you perform your IDD-related advocacy work as a medical student?

▼ Alabama (1) ... Outside of the United States or Canada (53)

11. In which state or country (if outside of the United States) did you perform your IDD-related advocacy work as a resident/fellow?

▼ Alabama (1) ... Outside of the United States or Canada (53)

Interview Guide

1. What motivated you to pursue your work in IDD-related curriculum advocacy?

PROBE: You mentioned you had a family member with an IDD/have family experience with people with IDDs. What part of that experience motivated you to pursue IDD-related curriculum advocacy? How did your family member motivate you to pursue IDD-related curriculum advocacy?

PROBE: You mentioned being frustrated about medical education regarding people with IDD/upset about the way your medical education was structured regarding people with IDD. Can you tell me more about how this emotion motivated you to pursue IDD-related curriculum advocacy?  

PROBE: You mentioned a particular clinical/volunteering experience. What about this experience motivated you to pursue IDD-related curriculum advocacy?

2. What changes have been made as a result of your IDD-related curriculum advocacy? (i.e. elective courses, required curriculum sessions, awareness campaigns, research, community service events, etc.)

PROBE: You mentioned curriculum changes. Can you go into more detail about these? For Example: How many people does the curriculum reach? Is it required or optional? How many disciplines are involved in the curriculum? Who teaches the curriculum? Which students usually participate in the curriculum?

PROBE: You mentioned changing the culture at your school/changing students' views on people with IDD. Can you elaborate on that change? How have you seen evidence of this change?

PROBE: You mentioned increased community involvement/engagement. Can you elaborate on how community involvement/engagement has changed as a result of your advocacy? 

3. Explain any barriers or frustrations you have encountered during your IDD-related curriculum advocacy.

PROBE: You talked about institutional barriers such as [mention barrier(s)]. Can you elaborate on those barriers? 

PROBE: You mentioned having a difficult time identifying faculty mentors. What was challenging about this process? 

PROBE: You mentioned difficulty with obtaining administrator buy-in. Can you elaborate on the difficulties that you experienced? 

PROBE: You mentioned a lack of student/peer support for your advocacy. Can you elaborate on the challenges associated with getting other students to support your advocacy work?

4. How have you been able to overcome barriers to your IDD-related curriculum advocacy work?

PROBE: You mentioned that your faculty mentor helped you overcome barriers to IDD-related curriculum advocacy. What enabled them to help you with your IDD curriculum advocacy? What steps did they take to ensure your advocacy work was successful?

PROBE: You mentioned that your institution/administration helped you overcome barriers to IDD-related curriculum advocacy. Can you elaborate on what they did that reduced the barriers you were experiencing? What steps did they take to ensure your advocacy work was successful?

PROBE: You mentioned that community partners/individuals with IDD helped you achieve your curriculum goals. Can you elaborate on how they helped you overcome the barriers you were experiencing? 

PROBE: You mentioned that other students helped you overcome barriers. What steps did they take to help with your advocacy efforts? 

PROBE: You mentioned an affiliated student group. Can you elaborate on how this group helped you overcome barriers to IDD-related advocacy?

5. What are some ways that your IDD-related mentor can support you to overcome the barriers to IDD-related curriculum advocacy?

PROBE: You mentioned that your mentor helped you overcome barriers to your IDD-related curriculum advocacy. Can you elaborate on the things that they did to help you?

PROBE: You mentioned that you did not have a supportive IDD-related mentor.
If they have a mentor: How could your mentor be more supportive?
PROBE: If they do not have a mentor: Why do you not have a supportive IDD-related faculty mentor? If you could have one, what would you want them to do to support you?

6. What are some ways that your institution can support you to overcome the barriers to IDD-related curriculum advocacy?

PROBE: You mentioned that your institution helped you overcome barriers to your IDD-related curriculum advocacy. Can you elaborate on the things that they did to help you? 

PROBE: You previously mentioned that you experienced institutional barriers to curriculum advocacy. Can you elaborate on specific ways that your institution could have better supported you? 

PROBE: You mentioned an affiliated student group. Can you elaborate on how this group helped you overcome barriers to IDD-related advocacy?

7. Do you have a plan in place to sustain your advocacy work after you leave your institution?

If YES: Please tell me more about that plan (who is involved, what it entails, etc.). Do you think it will be successful? Why or why not? 

If NO: Why do you not have a plan in place? 

PROBE: You mentioned you experienced challenges when trying to develop a sustainability plan. Can you elaborate on those barriers?

8. What do you wish your [medical school, residency, or fellowship program] did to enhance training related to intellectual disabilities?

[If the interviewee is a resident/fellow and performed curriculum advocacy as a medical student and during residency/fellowship] How has your advocacy experience differed as a medical student and as a resident? 

[If the interviewee is a resident/fellow and only performed advocacy work as a resident/fellow] How do you think your experience differs from a medical student performing IDD-related curriculum advocacy?

PROBE: You mentioned that your personal growth/development influenced your advocacy experience. Can you elaborate on this? 


PROBE: You talked about being more successful as a resident/fellow than a medical student. Can you elaborate on why you think you had more success as a resident? 

PROBE: You talked about being less successful as a resident/fellow than a medical student. Can you elaborate on why you think you had less success as a resident?

PROBE: You talked about having more free time/autonomy as a resident/fellow. How did this help you in your advocacy? How do you think we can help medical students be more successful advocates?
